# Disparities in EOL Care by Dementia Status and Race

**DOI:** 10.1093/geroni/igaa057.1527

**Published:** 2020-12-16

**Authors:** Joann Reinhardt

**Affiliations:** The New Jewish Home, New York, New York, United States

## Abstract

Prior research shows that minority and dementia status are associated with suboptimal end-of-life (EOL) care quality; care that is more aggressive, invasive, and futile. We conducted a retrospective study of EOL care for 300 decedents of varied race/ethnicity in a skilled nursing facility. The purpose of this secondary analysis was to test whether the EOL experience (medical orders in place, treatments, distressing symptoms, discussions with providers) differed by dementia status for different race/ethnic groups (Black, White, Hispanic). Chi-square tests were used to examine the relation between these four sets of EOL variables and dementia status (yes/no) separately for the three groups. Findings showed that for White decedents, PWD were less likely to have had a DNR or a DNI discussion with a provider in the nursing home. Also for White decedents, PWD were less likely to have had shortness of breath or pain. For Black decedents, PWD were more likely to have a DNR order. Also, for Black decedents, PWD were less likely to have been hospitalized. For Hispanic decedents, EOL variables and dementia status were not significantly associated. Overall, findings showed differences by race/ethnic groups in EOL experience based on dementia status. Black decedents with dementia were more likely to have escaped the acute care default. Findings for White decedents with dementia were mixed for aggressive versus comfort care. The EOL experience did not differ by dementia status for Hispanic decedents. Thus, efforts to promote positive EOL care for persons with dementia need to account for differences by race/ethnicity.

